# Rapid development of motion-streak coding in the mouse visual cortex

**DOI:** 10.1016/j.isci.2022.105778

**Published:** 2022-12-09

**Authors:** Manavu Tohmi, Jianhua Cang

**Affiliations:** 1 Department of Biology, University of Virginia, Charlottesville, VA 22904, USA; 2 Department of Psychology, University of Virginia, Charlottesville, VA 22904, USA

**Keywords:** Biological sciences, Neuroscience, Developmental neuroscience, Sensory neuroscience

## Abstract

Despite its importance, the development of higher visual areas (HVAs) at the cellular resolution remains largely unknown. Here, we conducted 2-photon calcium imaging of mouse HVAs lateromedial (LM) and anterolateral (AL) and V1 to observe developmental changes in visual response properties. HVA neurons showed selectivity for orientations and directions similar to V1 neurons at eye opening, which became sharper in the following weeks. Neurons in all areas over all developmental stages tended to respond selectively to dots moving along an axis perpendicular to their preferred orientation at slow speeds, suggesting a certain level of conventional motion coding already at eye opening. In contrast, at high speeds, many neurons responded to dots moving along the axis parallel to the preferred orientation in older animals but rarely after eye opening, indicating a lack of motion-streak coding in the earlier stage. Together, our results uncover the development of visual properties in HVAs.

## Highlights

• Developmental changes in visual response properties of mouse HVAs are observed
• HVA cells are orientation selective with lower OSI immediately after eye opening
• OSI of HVA neurons rapidly increases within one week after eye opening
• Motion-streak coding of visual cortical neurons develops rapidly after eye opening

## Graphical abstract

**Figure:**
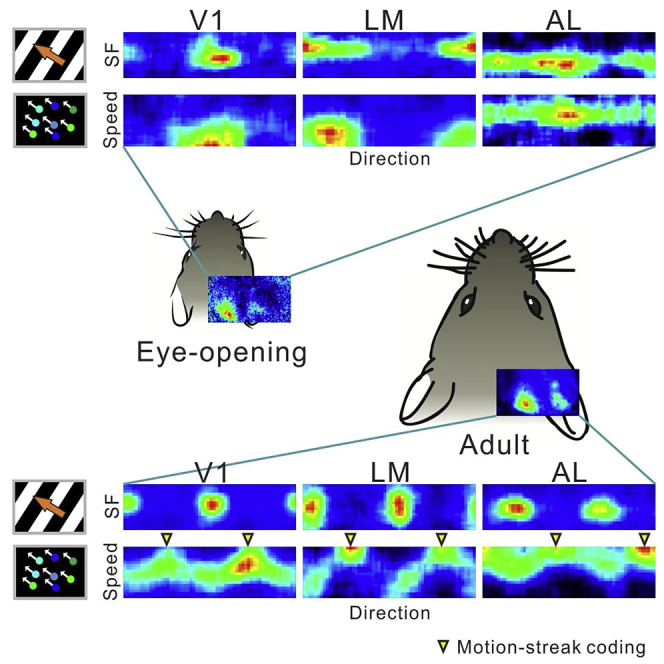

## Introduction

Visual cortex is composed of primary visual cortex (V1) and multiple higher visual areas (HVAs) which have different visual properties.^1^^,^^2^^,^^3^^,^^4^ The development of V1, but not HVAs, has been intensely studied. Previous studies have demonstrated developmental changes of various visual properties of V1 neurons, such as ocular dominance,^5^^,^^6^^,^^7^ orientation and direction selectivity,^8^^,^^9^^,^^10^ and binocular matching of orientation preference.^11^^,^^12^^,^^13^^,^^14^ Mouse V1 neurons are already orientation selective at eye opening, and their orientation tuning refines in the weeks afterward.^8^^,^^9^^,^^10^ The acquisition of orientation selectivity and its refinement seems to precede the developmental changes of other visual properties.^6^^,^^7^^,^^8^^,^^12^^,^^15^

Individual HVA areas have different levels of orientation and direction selectivity, speed tuning,^1^^,^^2^^,^^3^ and motion streak coding.^4^ How HVAs acquire their own functions during the very early developmental stages is an important and largely unanswered question. Only two studies have so far been conducted on mouse HVAs right after eye opening. Smith et al.,^16^ performed intrinsic signal imaging and found that the developmental time course was different for visual areas belonging to the dorsal or ventral streams. Murakami et al.,^17^ used wide-field imaging of transgenic mice expressing GCaMP6 and revealed that the functional segregation among HVAs was immature at eye opening. Since both studies used imaging of population activities at eye opening (the Smith et al.^16^ study did perform cellular imaging at later developmental stages), the response properties of individual neurons at this age are still unknown.

We recently discovered that motion streak information is encoded by neurons in mouse visual areas, including V1, LM, and AL.^4^ Visual cortical neurons tend to prefer motion direction along the axis perpendicular to their preferred orientation at slow speeds (conventional motion coding), whereas at high speed, many neurons tend to respond to visual stimulus moving along the axis parallel to their preferred orientation (motion-streak coding). Such motion streak responses are thought to provide useful information about object’s moving direction.^18^^,^^19^^,^^20^^,^^21^ Little is known about how visual cortical neurons develop motion-streak coding.

The aim of this study was to elucidate the visual response properties of individual neurons in mouse HVAs during postnatal development. We conducted 2-photon calcium imaging^22^ on V1 and higher visual area lateromedial (LM) and anterolateral (AL)^23^ at various developmental stages from right after eye opening to older than postnatal 4 weeks. Our experiments reveal the developmental profile of visual properties such as orientation/direction selectivity, conventional motion coding, and motion-streak coding.

## Results

**Figure 1:**
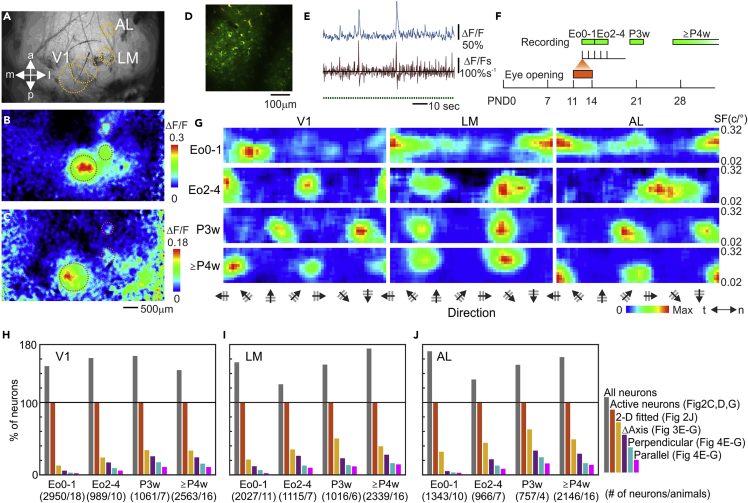
Two-photon imaging of neuronal responses in mouse V1 and higher visual areas during development (A–C) Autofluorescence imaging of visual cortical areas. (A) Non-processed autofluorescence image of cortical surface on the day of eye opening. (B) and (C) Images of autofluorescence signal changes evoked by drifting gratings placed at different visual field positions, 45° azimuth in (B) and 90° azimuth in (C). Dashed ellipses represent corresponding locations in the 3 images. See Figures S1A–S1C for autofluorescence imaging of adult mice. (D) An image of area AL at eye opening under 2-photon microscope. Green: cal-520 signal; red: SR-101 stained astrocytes. See Figure S1D for an adult mouse. (E) Calcium transients during visual stimulation. Top: raw calcium signal of an AL neuron at eye opening. Middle: running-averaged and differentiated signal of the top trace (see STAR Methods), where positive areas during visual stimulation were quantified as responses. Bottom: durations of visual stimulation are represented by green bars. (F) Experiment schedule. Mice opened eyes at PND11-14 (orange bar). They were imaged (green bars) at 0–1 day (Eo0-1) and 2–4 days (Eo2-4) after eye opening, as well as at Postnatal week 3 (P3w), and after week 4 (≥P4w). (G) Examples of visual responses of V1, LM, and AL neurons at different developmental stages. Tuned responses for stimulus direction/orientation (x axis, t: temporal, n: nasal) and spatial frequency (SF, y axis, logarithmically plotted) are seen as early as on the day of eye opening. See Figure S1E for other examples. (H–J) Populations of neurons used for individual analyses were plotted for the three areas. All proportions were normalized to the number of active- (see Figures S1F–S1I) and responsive-(Figure S1L) neurons in V1 (H), LM (I), and AL (J) at different development stages, which were used for analysis in Figures 2C, 2D, and 2G. See Figures S1F–S1P and Method for criteria.

V1, LM, and AL were identified by wide-field imaging of autofluorescent signals in mice of different ages^3^^,^^4^^,^^24^^,^^25^ (Figures 1A–1C and S1A–S1C). Visual stimulus placed in the left visual space evoked signal changes of the autofluorescence in the contralateral visual cortex on the day of eye opening (Eo; Figures 1B and 1C)^16^^,^^17^ as well as in adult mice (Figures S1B and S1C).^3^^,^^4^ According to retinotopic structures of individual visual areas,^3^^,^^4^^,^^23^ regions of V1, LM, and AL were determined and then injected with a calcium indicator cal-520AM (Figures 1D and S1D) following craniotomy. Recordings were made (Figure 1E) in mice at various developmental stages (Figure 1F), within 48 h (Eo0-1) or 2–4 days (Eo2-4) after eye opening which occurred on postnatal days 11–14 (orange bar), at the postnatal 3 weeks (P3w), or older than postnatal 4 weeks (≥P4w). Different animals were used to measure the responses at different ages, with 1 or 2 imaging sessions for each mouse on the same day of surgery. We used drifting gratings to determine the orientation/direction preference of individual neurons and used moving dots to reveal the relationship between cells’ preferred orientation and motion axis. Stimulus parameters and data analyses followed our recent studies of adult HVAs.^4^ To analyze various visual response properties, we used various criteria to select neurons (see STAR Methods), and numbers of neurons selected were shown in Figures 1H–1J.

### Development of orientation/direction selectivity

**Figure 2:**
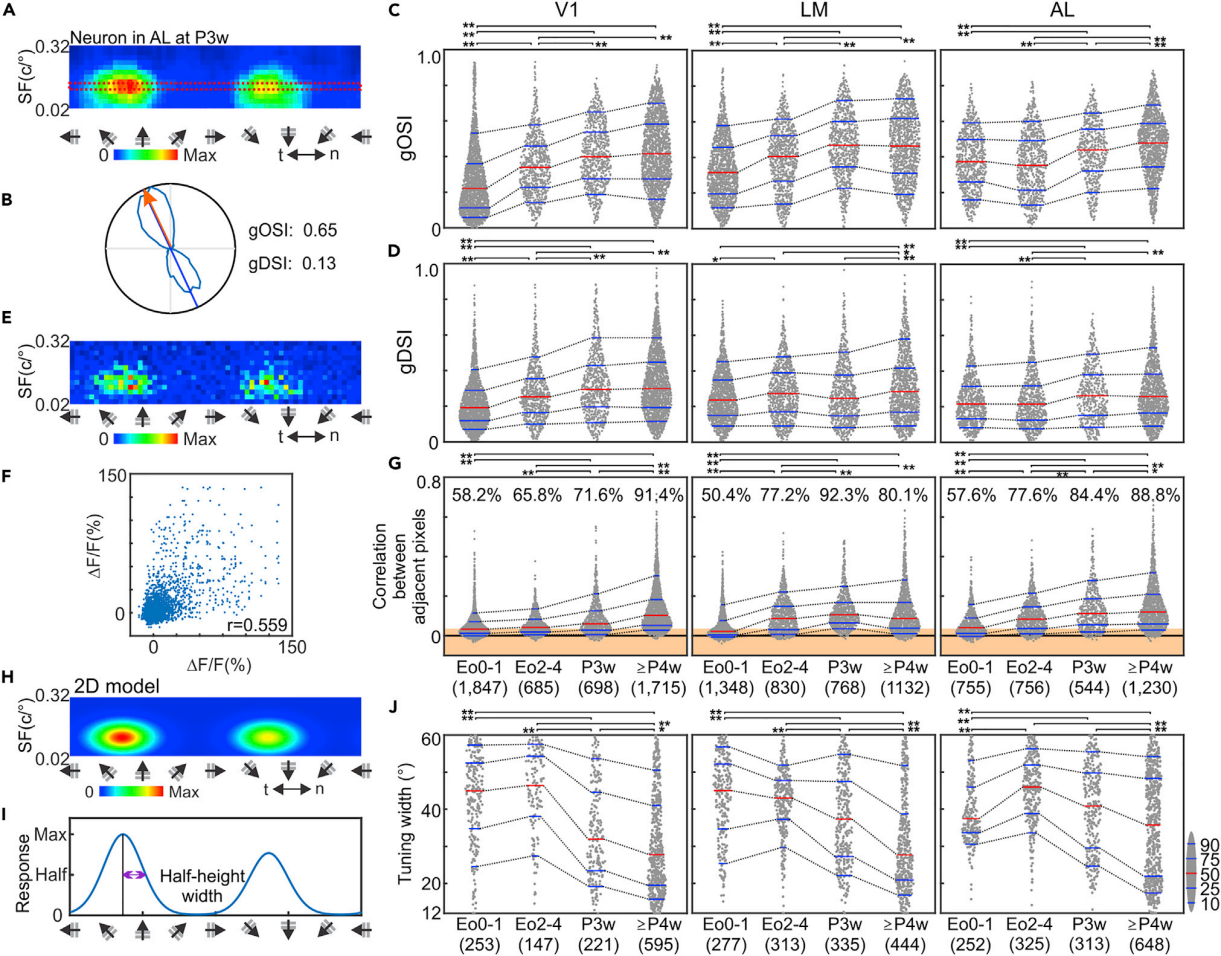
Development of orientation and direction selectivity of visual cortical neurons after eye opening (A and B) Quantification of Orientation and Direction selectivity. (A) From the filtered response map, the row containing the max response (the red dashed box) is used to calculate tuning indices to drifting gratings. (B) A radar plot of the example neuron’s tuning curve (light blue line). Preferred motion axis of drifting gratings (blue straight line), preferred direction (orange arrow), and gOSI/gDSI are calculated by the vector-sum method. (C and D) gOSIs (C) and gDSIs (D) of neurons responsive to drifting gratings in V1 (left), LM (middle), and AL (right) at different development stages. (E and F) Quantification of response reliability. (E) Non-filtered response map of (A). (F) Correlations of signal intensities between pairs of adjacent pixels (4 sides for each pixel) in response map in (E). The correlation coefficient is used as a measure of response reliability. (G) Response reliability increases in all areas over development. The light orange area represents the statistically non-significant correlation between adjacent pixels (p > 0.05, r < 0.036, 3,000 pairs, Pearson’s test). The value above each plot represents the proportion of neurons with a statistically significant correlation. (H and I) Quantification using 2D fitting. (H) A 2D-model fitted to the non-filtered response map in (E). (I) The direction-tuning curve of the fitted 2D model at the row containing the maximum. Purple double-headed arrow represents the half-width at half height. (J) Tuning widths of individual neurons evaluated by 2D model fitting. In C, D, G, and J, each dot represents a single neuron. Blue bars: 10, 25, 75, and 90 percentiles of the entire populations. Red bars: median. The numbers beneath charts represent the number of neurons. One-way ANOVA for ages, p < 0.001 for all areas in (C, D, G, and J). See Figures S2A–S2D for individual animals, Figures S2E–S2H for analysis of recording depth, and Figures S2I–S2N for SF tuning.

Some V1 neurons were already selective for specific visual stimulus at eye opening conditions as previously reported^8^^,^^9^^,^^10^ (Figures 1G and S1E, top left). We also found that some neurons in LM and AL were already selective for specific visual stimulus conditions at Eo0-1 when the animals had little visual experiences (Figure 1G, top second, and third panels, and S1E, left column). We calculated a global Orientation Selectivity Index (gOSI; Figures 2A–2C) to observe developmental changes in the orientation selectivity of these visual areas. The results demonstrated that gOSI of neurons in all areas around eye opening tended to be lower, and gradually increased over development (Figure 2C, over development, p < 0.0001 for all areas, one-way ANOVA; see Figures S1F–S1P and STAR Methods for cell-selection criteria, and proportion of neurons selected for each analysis is shown in Figures 1H–1J). However, there were differences in the time course of improvement of gOSI between areas. Neurons in V1 and LM increased their gOSI between Eo0-1 and Eo2-4 (p < 0.0001, Tukey HSD post-hoc test), but not those in AL (p = 0.204). On the hand, AL neurons increased gOSI after Eo2-4 and continue to improve after P3w (P3w vs ≥ P4w, p < 0.0001), while there was no significant change in V1 (p = 0.228) and AL (p = 0.878) after P3w. Developmental changes in direction selectivity were also evaluated by calculating a global Direction Selectivity Index (gDSI) of responsive neurons, which showed that direction selectivity slightly increased during development in all areas (Figure 2D; over development, p < 0.0001 for all areas, one-way ANOVA; all combination of Eo0-1 vs other ages, p < 0.005 for all areas except for LM at P3w, p = 0.546, and AL at Eo2-4, p = 0.980, Tukey HSD post-hoc test).

It was possible that the low gOSIs and gDSIs at eye opening were at least in part attributed to low response reliabilities. We could not determine trial-trial reliability directly because of the low number of repeats that were necessary to achieve a fine resolution of stimulus variables. Instead, we compared the response intensities between all pairs of adjacent pixels (4 sides) in the non-filtered response map (Figure 2E), which would be similar if the neuron responded reliably. We calculated the correlation coefficient for individual neurons (Figures 2E and 2F), which showed gradual increases after eye opening in all areas (Figure 2G), indicating increasing response reliability to certain visual stimuli during development. We also calculated the percentage of neurons that met the criteria of p < 0.05 by Pearson’s test (i.e., r > 0.036, 3,000 pairs, those above the light-orange areas in Figure 2G, see Figure S1P for correlation between response reliability and selectivity for direction and SF). About 40–50% of “active cells” in all visual areas were not reliably responsive by this statistical criterion at Eo0-1, whereas about 90% of the active cells (Figures 1H–1J) responded reliably after P4w.

To study orientation selectivity development further, we analyzed neurons with high response reliability (adjacent pixel correlation, p < 0.05, Pearson’s test) and well-defined tuning curves (for instance, Figures 1G, 2A, and S1E). Specifically, we fitted individual response maps using a 2D model, which is a combination of a double von Mises curve for directions and a Gaussian curve for spatial frequency (SF) (Figure 2H). Neurons with a correlation coefficient between non-filtered map and 2D-model >0.75 were included in this analysis, where the half-width at half-max of von Mises curves (Figure 2I, the purple double-headed arrow) was used to quantify orientation tuning width (Figure 2J). At Eo0-4, tuning curves of most of these selected neurons were wider than 40° (68.0%, 63.9%, and 56.8% for V1, LM, and AL) and only small portions had sharp tuning less than 30° of width (15.3%, 14.2%, and 6.6 for V1, LM, and AL). Orientation tuning became shaper with development, when much more neurons had orientation-tuning curves narrower than 30° (≥P4w, 54.0%, 47.0%, and 36.1% for V1, LM, and AL; over ages, p < 0.0001 for all areas, Eo0-1 and Eo2-4 combined, one-way ANOVA, Tukey HSD post-hoc test). Together, these results indicate that after eye opening more V1, LM, and AL neurons became reliably responsive and selective for stimulus orientation and that the selective neurons became more sharply tuned (see Figures S2I–S2N for SF tuning).

Finally, we repeated the above analyses based on individual animals (Figures S2A–S2D and Table S1) and separated neurons according to depth (Figures S2E–S2H). All major developmental changes were confirmed by these analyses (note that the increase in gDSI of LM neurons between Eo0-4 and ≥P3w was not confirmed in data from shallow sites in Figure S2F).

### Development of conventional and motion-streak coding

**Figure 3:**
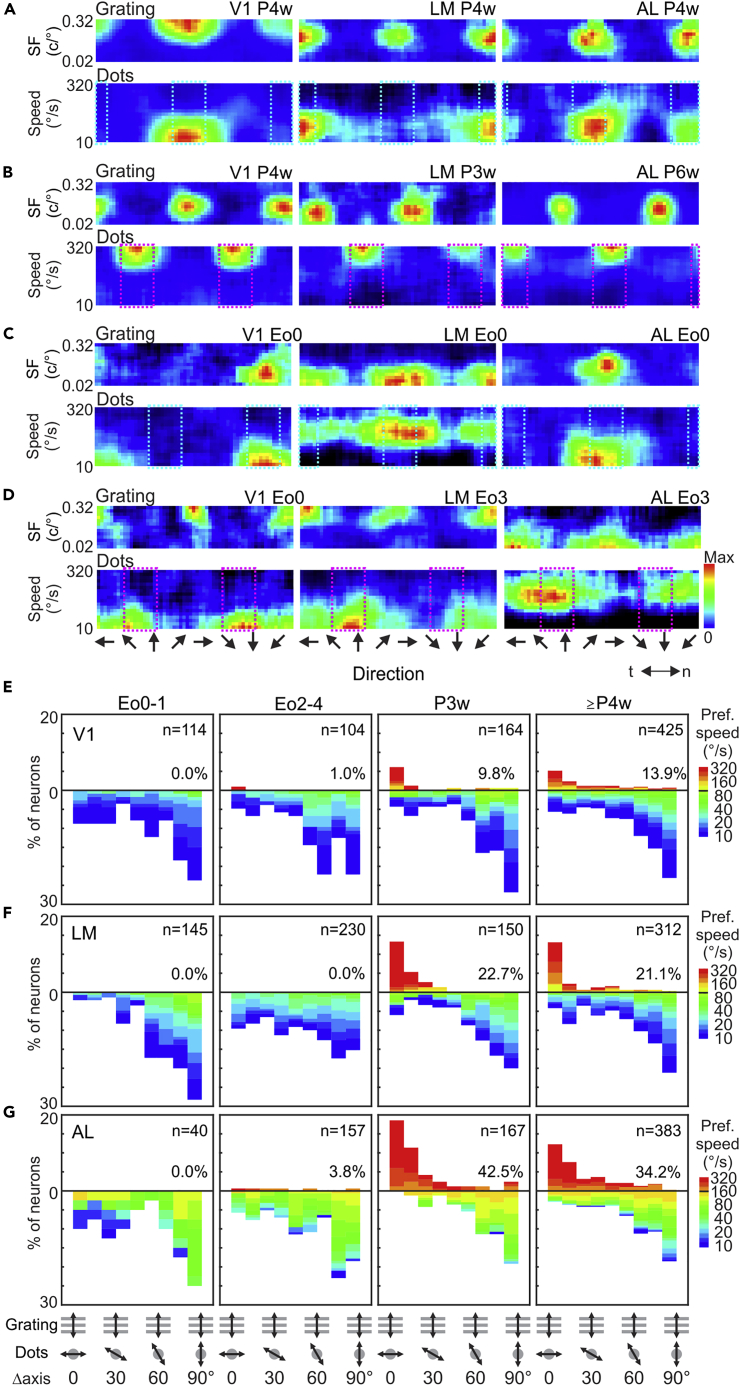
Development of motion streak encoding in mouse cortical areas (A–D) Example responses to drifting gratings (top) and moving dots (bottom) of neurons in V1 (left), LM (middle), and AL (right), at age of ≥P3w (A and B) or Eo0-4 (C and D). Some neurons (e.g., A and C) were tuned to the same motion axis (light-blue dashed boxes) in response to gratings and moving dots, whereas others (B and D) were tuned to orthogonal motion axes (pink dashed boxes) to the two types of stimuli. (E–G) Distribution of the difference between preferred orientation of drifting gratings and motion axis of moving dots (Δaxis) of V1 (E), LM (F), and AL (G) at different ages. Colors represent the preferred speed. Population of neurons with preferred speeds higher or lower than the invert speed (100.8°/s for V1 and LM, and 160°/s for AL, see Figure S3A) are plotted upward or downward, respectively. Percentage values in each panel represent the proportion of neurons with preferred speeds higher than the invert speed. See Figures S3B–S3G for the analysis of recording depth.

To study the development of conventional motion processing and motion-streak coding, we used random dots moving in various directions with a range of speeds in addition to drifting gratings. In later development stages (≥P3w), in the low-speed range, neurons in all three areas tended to prefer directions of moving dots similar to the preferred motion-axis of gratings, i.e., the axis perpendicular to the preferred orientation (“perpendicular axis,” Figure 3A, light-blue dashed boxes), indicating conventional motion coding. In the high-speed range, many neurons preferred motion axes parallel to the preferred orientation (Figure 3B, pink dashed boxes), indicating motion-streak coding. These results are similar to what was observed in awake adult mice in our previous study.^4^ In the early developmental stages (Eo0-4), many neurons in all three areas preferred the perpendicular axis (Figure 3C, light-blue dashed boxes) or the parallel axis (Figure 3D, pink dashed boxes) at the low-speed range. To quantify this, we calculated the angle difference between the preferred grating orientation and the preferred moving dot axis, namely “Δaxis,” and plotted its distribution at different developmental stages (Figures 3E–3G). We also determined the “invert speed” for each area based on the data from later developmental stages, above which the proportion of neurons preferring the parallel axis became dominant over the proportion of neurons preferring the perpendicular axis (≥P3w; Figure S3A, 100.8 for V1 and LM, 160°/s for AL, respectively). In the speed range lower than the invert speed (downward bars in Figures 3E–3G), neurons tended to prefer the perpendicular axis (Δaxis >60°, >50% of neurons for all areas at all ages except for LM at Eo2-4, 45.2%) over the parallel axis (Δaxis <30°, 5.51–26.3%) in all areas over development (parallel vs perpendicular, p < 0.001 for all areas and all ages except for AL at Eo0-1, p = 0.117, chi-square test). This suggests that V1, LM, and AL neurons at eye opening already show conventional motion coding. On the other hand, very few neurons preferred speeds higher than the invert speed at Eo0-4 (0.0–3.8%), and their proportion increased in the following week (9.8–42.5% for P3w and ≥P4w, upward bars in Figures 3E–3G). By P3w and P4w, most of these high-speed cells preferred the parallel axis (neurons with Δaxis <30°, 62.7–94.1%; parallel vs perpendicular, p < 0.001 for all areas and all ages, chi-square test). These results thus indicate that motion-streak coding develops rapidly in all three visual areas during this period (see Figures S3B–S3G for analysis of recording depth).

**Figure 4:**
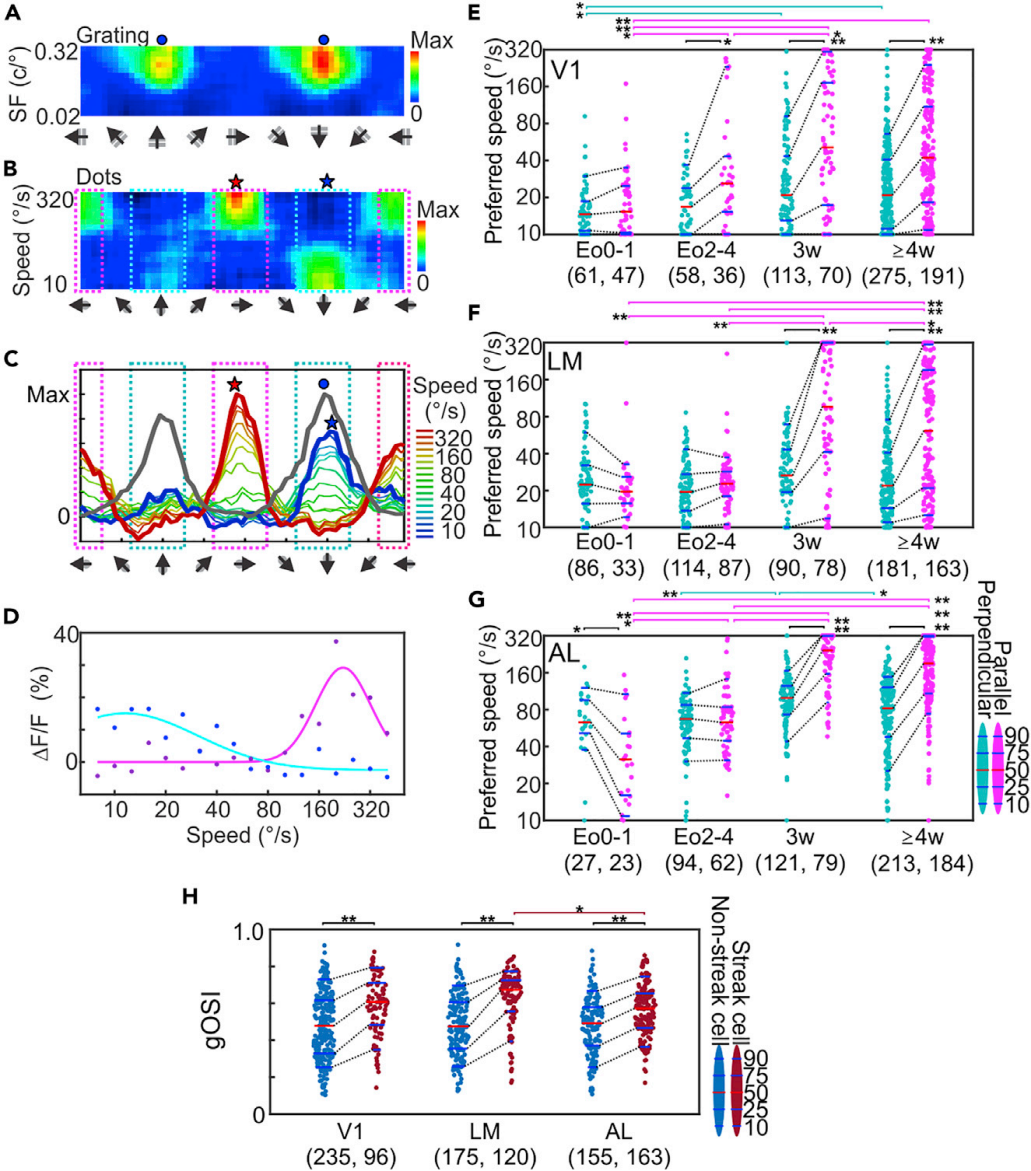
Development of speed tuning along perpendicular- or parallel-axis of preferred orientation (A and B) Response maps for drifting gratings (A) and moving dots (B) of an LM-neuron at P4w, which showed conventional motion coding at slow speeds and motion-streak coding at high speeds. (C) Direction-tuning curves for moving dots, correspond to individual rows of direction-tuning curves in the response map in (B). Blue circles in (A) and (C): Preferred motion-axis of drift grating. Blue and red stars: The directions at which the largest peak of direction curves (rows) within 30° from perpendicular-(blue star) and parallel-(red star) axes were found. Light-blue dashed boxes in (B) and (C): directions within 30° from the preferred motion axis (perpendicular axis). Pink dashed boxes: directions within 30° from the preferred orientation (parallel axis). See Figure S4A for other. (D) Gaussian fitting to determine preferred speeds. The responses at different speeds at the perpendicular-(blue dots) and parallel-(purple dots) axis are plotted and fitted by Gaussian curves. See Figure S4B for data selection. (E–G) Preferred speeds of moving dots along perpendicular (light blue) or parallel (pink) axis of V1 (E), LM (F), AL (G) neurons at different developmental stages. Two-way ANOVA for axis x ages: axis: p < 0.0001, among ages: p < 0.0001, interaction: p < 0.0001. Black bracket: perpendicular vs parallel. Light blue (or pink) bracket: comparison of preferred speeds at perpendicular-(or parallel) axis between ages. (H) gOSI of streak cells (brown) and non-streak cells (blue) of V1, LM, and AL at age ≥ P3w. Cells in E-G with preferred speed at parallel axis higher than the invert speed (100.8°/s for V1 and LM, and 160°/s for AL, see Figure S3A) were defined as streak cells and other cells in (E–G) as non-streak cells. Two-way ANOVA for non-streak vs streak x area: non-streak vs streak: p < 0.0001, areas: p = 0.035, interaction: p = 0.085. In (E–H), each dot represents a single neuron. ∗: p < 0.05, ∗∗: p < 0.001, Tukey post-hoc test.

A number of neurons, the so-called transition cells, show both conventional motion-coding and motion-streak coding.^4^^,^^26^^,^^27^ Such neurons responded well to the “perpendicular axis” at slow speeds (Figures 4B and 4C, light-blue dashed boxes; Figures 4A and 4C, blue dots: preferred motion-axis direction, see Figure S4A for other examples), but the “parallel axis” at high speeds (Figures 4B and 4C, pink dashed box). It was possible that the lack of motion-streak coding at eye opening in Figures 3E–3G was due to smaller parallel axis responses hidden under larger perpendicular axis responses of the same cells, thus not selected for analysis. To test this, we analyzed moving dot tuning curves separately at individual speeds for each cell. In this analysis, we first determined the largest response within 30° of the perpendicular axis (Figures 4B and 4C, light-blue dashed boxes) and then calculated the preferred speed at the corresponding direction by Gaussian fitting (Figure 4D). The same was done for the parallel axis (Figures 4B and 4C, pink dashed boxes). At Eo0-4, the preferred speed at the parallel axis was roughly similar to those at the perpendicular axis in all areas, although there were slight differences in V1 at Eo2-4 and AL at Eo0-1 (Figures 4E–4G). However, the preferred speed at the parallel axis rapidly increased and became much higher than that at the perpendicular axis by P3w. At the perpendicular axis, the median of preferred speeds in V1, LM and AL slightly increased by 40.6%, 16.6%, and 37.2% from Eo0-4 to ≥ P3w, while those at the parallel axis increased dramatically by 126%, 266%, and 280% (Figures 4E–4G, Eo0-1 and Eo2-4 are combined for statistical comparisons with P3w and ≥P4w combined, axis x Eo0-4 vs ≥ P3w, age: p < 0.0001, Eo0-4 vs ≥ P3w: p < 0.0001, interaction: p < 0.0001 for all areas, two-way ANOVA; perpendicular axis Eo0-4 vs ≥ P3w: p < 0.001, p = 0.029, 0.002 for V1, LM, and AL, parallel axis: p < 0.0001 for all areas, Tukey HSD post-hoc test). We demonstrated the above analyses based on individual animals (Figures S4C–S4E and Table S1) and all major developmental changes were confirmed (note that the difference between axes at Eo0-4 but not at Eo0-1 in V1 was observed). Together, these results indicate that speed tuning of motion streak coding in these areas develops rapidly after eye opening, consistent with the analysis in Figures 3E–3G. The results also demonstrated that the preferred speed of AL neurons at the perpendicular axis, i.e., the preferred speed of conventional motion coding, was higher than those of V1 and LM neurons over all ages (p < 0.0001 for all ages and AL vs V1 or LM, following two-way ANOVA of age x area, age: p < 0.0001, area: p < 0.0001, interaction: p = 0.414). This suggests that the acquisition of the faster speed tuning of AL neurons^1^^,^^2^^,^^3^^,^^4^^,^^17^ most likely does not require visual experience.

Finally, we examined the relationship between motion-streak coding and orientation selectivity in later developmental stages (≥P3w). Cells with the preferred speed at the parallel axis higher than the invert speed were considered as “streak cells,” and the orientation selectivity for grating pattern was compared between streak cells and non-streak cells (Figure 4H). Streak cells in all areas showed higher gOSI than non-streak cells (streak vs non-streak: p < 0.0001 for all areas, Tukey HSD post-hoc test following two-way ANOVA of streak vs non-streak x area, streak vs non-streak: p < 0.0001, areas: p = 0.035, interaction: 0.085), suggesting a potential relationship between the development of streak coding and refinement of orientation selectivity.

## Discussion

In this study, we found that HVA neurons at eye opening showed preference for a specific orientation similar to V1 neurons. This confirms and extends previous results that neurons in mouse V1 at eye opening are already selective to specific orientation,^8^^,^^9^^,^^10^ and suggest that the establishment of orientation selectivity of HVA does not require visual experience. Our study does not completely exclude the possibility of short visual exposure by the pups in their home cage or during wide-field imaging before two-photon imaging. However, a previous study reported that short-term visual experience did not cause significant changes in visual properties of V1.^8^ Experience-independent mechanisms to establish orientation preference of V1 neurons are still largely unknown. Spontaneous activities in the visual system, such as synchronous activities in the visual cortex^28^^,^^29^^,^^30^ or retinal waves,^31^^,^^32^^,^^33^ could shape the receptive field of V1 neurons through activity-dependent synaptic plasticity,^31^^,^^33^^,^^34^^,^^35^ whereas genetic mechanisms establishing orientation selectivity are also possible.^36^^,^^37^^,^^38^^,^^39^ Whatever mechanisms determine V1 orientation selectivity presumably also contribute to the development of orientation selectivity in HVAs.

We demonstrated a similar process in HVAs, where the orientation tuning rapidly sharpens during the period between Eo4 (roughly P15-18) and P3w (∼P21), and still improves afterward. Refinement of neural circuits in V1 and LM occurs simultaneously in the early developmental stage. This is somewhat surprising because activities of HVAs are thought to depend on direct and/or indirect inputs from V1^3^^,^^40^^,^^41^ and development of HVA visual responses in other mammals tend to be preceded by that of V1.^42^^,^^43^^,^^44^^,^^45^^,^^46^ While feedback projections from HVA to V1 are still immature at eye opening, feedforward projections from V1 to LM are already close to that in the adult mouse.^47^^,^^48^ A large number of convergent inputs from V1 to LM neurons may compensate the immature visual properties of individual V1 neurons. In addition, recent studies demonstrated the importance of inputs from the superior colliculus via LP thalamic nucleus on HVA visual properties.^3^^,^^40^^,^^41^^,^^49^ These inputs could play an important role in establishing visual properties of HVAs around eye opening. On the other hand, AL neurons showed a slightly delayed development of orientation tuning. Different developmental time courses of innervation from V1 to different HVAs^50^^,^^51^ may lead to different maturation of visual properties between areas.

We also demonstrated that V1 and HVAs show conventional motion coding at eye opening. Since the proportion of neurons selected for the analysis was much smaller at eye opening than in later developmental stages (Figures 1H–1J), it is possible that more neurons develop conventional motion-coding after vision onset. However, Murakami et al.^17^ demonstrated that temporal frequency tuning of population activities of LM and AL neurons at eye opening was almost similar to those of adult mice, supporting the notion of largely mature conventional motion process right after vision onset. Contrary to conventional motion coding, neurons in all areas do not encode motion streak at eye opening, and motion-streak coding rapidly develops in the week after. Motion streak neurons are activated by visual objects moving along their elongated receptive fields. It is known that orientation selectivity of V1 neurons increases after eye opening as the shape of the receptive field becomes elongated.^10^ Therefore, a possible explanation for the lack of motion-streak coding at eye opening is the immature receptive field that is not elongated enough and/or low response reliability that would lead to insufficient temporal and spatial summation. Consistent with this idea, orientation tuning is rapidly sharpened at the time when neurons develop motion-streak coding (Figure 2J), and streak cells tend to have higher orientation selectivity (Figure 4H). In addition, earlier visual structures including the retina and dLGN could still be too immature to code motion streaks at the early development stage, although this is less likely because of the level of conventional motion coding seen at eye opening.

Finally, we note that there are slight differences in the exact percentage of neurons encoding motion streaks between ≥ P4w (Figures 3E–3G) in this study and adult mice in our previous report.^4^ For instance, in the previous study, more V1 neurons preferred motion streak coding to conventional motion coding, and more AL neurons showed conventional motion coding in high-speed range. The most likely factor causing this difference is anesthesia. In awake condition (the case in the previous study), neural interactions between cells which can integrate two types of motion information are more active and leading cells to encode various visual information such as motion and contours in a wide speed range.^18^

### Limitations of the study

Due to technical difficulties, our recordings were made under anesthesia. Therefore, the visual response properties observed in this study may differ from those in the awake state. It is also difficult to match the depth of anesthesia level among age groups because of possible differences in sensitivity to anesthesia. The lower responsiveness of neurons in young mice may potentially be due to differences in sensitivity to anesthesia. In addition, mice at Eo0-1 have had visual experiences for several hours before recording neural activities, and we had to identify visual areas by macroimaging with visual stimuli before 2-photon imaging. Thus, it was possible that the short-term visual experience may cause changes in visual response properties in these young mice.

## STAR★Methods

### Key resources table

**Table:** 

REAGENT or RESOURCE	SOURCE	IDENTIFIER
**Bacterial and virus strains**		

AAV1-Syn-GCaMP6f viral vector	addgene	100837

**Chemicals, peptides, and recombinant proteins**		

Carprofen	Zoetis	141-199
Chlorprothixene hydrochloride	Sigma	C1671
Vetbond	3M	70200742529
Metabond	Parkell	S380
Isoflurane	Covetrus	1169567761

**Experimental models: Organisms/strains**		

C57BL/6	bread at lead contact lab	N/A

**Software and algorithms**		

Psychophysics Toolbox	Psychtoolbox	RRID: SCR_002881http://psychtoolbox.org/docs/Psychtoolbox
MATLAB 2018a	MathWorks	RRID: SCR_001622https://www.mathworks.com/products/matlab.html
LabVIEW 2017	National Instruments	RRID: SCR_014325 https://www.ni.com/en-us/shop/labview.html

**Other**		

Microinjector	Drummond Scientific	Nanoject II
CCD camera	Bitran	BU-61M
470 nm LED	Prizmatix	UHP-T-LA
LCD monitor	ViewSonic	VA951S
LCD monitor	MSI	Optix G24C
Temperature control	FHC	N/A
Head Holding Device	Narishige	SR-AM
Anesthesia Systems	Kent scientific	VetFlo

### Resource availability

#### Lead contact

Further information and requests for resources and reagents should be directed to and will be fulfilled by the Lead Contact, Manavu Tohmi (tohmim@gmail.com).

#### Materials availability

This study did not generate new unique reagents.

### Experimental model and subject details

All experimental procedures were approved by the University of Virginia Institutional Animal Care and Use Committee. Wild-type C57BL/6 mice of both sexes at various ages from postnatal day 11–120 were used. Two to five animals after weaning were housed per cage on a 12 h light/dark cycle. Around age of P10-14, pup’s eyes were checked every day to ascertain eye opening timing.

### Method details

#### Surgery

Mice were anesthetized with isoflurane (4% for induction, 0.1–0.4% for maintenance, in O_2_, 0.5 L/min; VetFlo, Kent scientific) followed by injection of chrolprothixene (2.5 mg/kg, i.m., Sigma) which reduces the concentration of isoflurane needed to keep a mice anesthetized. All surgeries and recordings were done under anesthesia. We checked the depth of anesthesia by observing the reflex to pinching a hind paw. Body temperature was maintained at 37°C by a heating pad equipped with a feedback control module monitored via a rectal thermoprobe (Frederick Haer) for mice older than postnatal days 18, or the heater pad was maintained at 37°C for younger mice. Skin was disinfected using alternating scrubs of 70% ethanol and removed to expose the skull. A plastic head holder made by 3-D printer (Ultimaker) was mounted on the skull using Metabond (Parkell) mixed with black ink to avoid reflections during imaging. The skull was immersed in saline to keep transparent. Wide-field autofluorescent imaging was performed transcranially to identify visual areas (details below). After wide-field imaging, craniotomy was performed to expose the areas of interest.

50 μg Cal-520 AM (AAT Bioquest) was dissolved in 4 mL DMSO + 20% pluronic-127 (invitrogen) and sonicated for 1 min. The dye was diluted with saline containing Sulforthodamine101 (SR-101, Sigma) to a final concentration of 500 μM cal-520 and 50 μM SR101. The solution was filtered with a centrifugal filter (UFC30HV, Millipore) and injected using a Nanoject II (Drummond Scientific) fitted with a glass pipette into several sites in each visual area corresponding to the visual field around 60° in azimuth and 30° in elevation by referring to response maps of wide-field autofluorescent imaging. The pipette was loaded with the solution, and inserted at a 45° angle. The solution was injected at 300 and 200 μm below surface in 9.2 nL volume x 6pulses, 20 s intervals. After injections, a 4 mm diameter glass coverslip was placed on the brain surface and sealed with Vetbond (3M) and Metabond (Parkell). Two-photon imaging experiments were performed more than 2 hours after dye injection. One or two sets of recordings in different visual areas of one mouse were made on the same day of the surgery. Different animals were used to measure the responses at different ages.

#### Wide-field autofluorescent imaging

Wide-field autofluorescent imaging and two-photon calcium imaging were performed as described previously.^4^ A binocular microscope (MVX10, Olympus) equipped with a 0.63 x, 0.15 NA objective (MV PLAPO, Olympus), an Olympus U-M49002XL filter cube (excitation filter: 470/40 nm, dichroic mirror: 495 nm high pass, emission filter: 525/50 nm) and a CCD-camera (BU-61M, Bitran), was used to transcranially record flavoprotein fluorescence signals. The brain surface was excited with a 470 nm LED (UHP-T-LA, Prizmatix) of ∼4.2 mW below the objective. Images (240 × 135 pixels) were recorded at 10 Hz with custom acquisition software (LabVIEW 2017, National Instruments).

#### Two-photon calcium imaging

The mice were head-fixed under a two-photon scanning microscope (Ultima Investigator, Bruker Nano Surface Division; RRID: SCR_017142). Imaging was performed with a Ti:sapphire laser (Chameleon Ultra 1, Coherent) at excitation wavelength of 920 nm for cal-520 using a 16x, 0.8 NA Nikon objective tilted to be perpendicular to the brain surface. Emitted signals from cal-520 (green) and SR-101 (red) were separated by a dichroic mirror into PMTs. Imaging data were acquired using the PrairieView software v5.4 with a resonant scanner at 2x optical zoom, resulting in a 412.2 × 412.2 μm field-of-view. Image resolution was 512 × 512 pixels and the acquisition rate was 30 Hz. Imaging was performed in the depth of 150–350 μm from the surface which presumably corresponded to the layer 2/3. To address effects of recording depth on our results, we separated data into two groups from sites above or under 280 μm from the surface. The comparisons are shown in Figures S2E–S2H and S3B–S3G, and indicate that recording depth did not change our conclusions of developmental changes.

#### Visual stimulus

All visual stimuli were generated with MATLAB Psychophysics toolbox.^52^ For wide-field autofluorescent imaging, visual stimuli were presented on an LCD monitor (ViewSonic, 19 inches, 60 Hz refresh rate, ∼50 cd/m^2^ mean luminance, gamma corrected). The distance from the eye to the monitor was 25 cm. To create retinotopic maps for identification of visual areas (Figures 1B, 1C, S1B, and S1C), the screen center was placed in various visual space between 30° and 90° across the azimuth and between 0° and 45° in elevation to stimulate various visual field. Moving dots of 2° in diameter and 10°/s in speed were presented in a Gaussian window (10° standard deviation) on black background for 2 s, followed by an 8 s black screen. Density was 0.015 dots/deg^2^. Color of each dot was set randomly in range of RGB (0,255,0) ∼ RGB (0,255,255) ∼ RGB (0,0,255). We used color dots, because they evoked stronger responses than white dots or black-white dots.^4^ Trials were repeated until fine images were acquired, typically 40 times for mice older than postnatal days 20, and >80 times for mice around eye opening.

For 2-photon imaging, visual stimuli were presented on an LCD monitor (MSI, 24 inches, 144 Hz refresh rate, curved surface, ∼50 cd/m^2^ mean luminance, gamma corrected). We measured its refresh speed with a photodiode (2 kHz sampling rate). When screen luminance was changed from the baseline (RGB: 0, 0, 0) to the max (RGB: 0, 255, 255), the time to reach 50%, 90%, and 99% of the max luminance was 1.5 ms, 4 ms, and 9 ms, respectively. When luminance was flipped from the max to the baseline, the time to 50%, 10%, 1% was 5 ms, 9.5 ms, and 15 ms. The screen was placed 25 cm away from the eye contralateral to the imaging site (the left eye) covering 92.7° × 64.3° (width x height) of the visual field. Usually, the monitor center was placed around 60° (45–75°) in azimuth and 30° (15–45) in elevation, since responsive regions of visual areas corresponding to this visual field were separated well from each other. The placement of the monitor center was adjusted according to the retinotopic maps created by wide-field autofluorescent imaging. We did not apply distortion compensation to visual stimuli since we could accurately place the monitor in the receptive field by referring autofluorescent response maps, and the receptive fields of most of cells were presumably located in the center part of the monitor.

Moving dots, and drifting grating were used for 2-photon imaging.

##### Drifting gratings

Full-screen square-wave drifting gratings (100% contrast, 3 Hz) with 60 different directions and 16 spatial frequencies (SF; 0.01–0.32 c/° with exponentially equal intervals) were presented for 667 ms followed by a gray full screen for 833 ms. The body of a young mouse is smaller and more transparent with less fur than that of adult, we could not eliminate visual stimulation light passing through the body completely, affecting calcium signals in low SF range in some recordings of young mice. Thus, we excluded data of responses to low SF (0.01–0.0159 c/°) for all analysis.

##### Moving dots

Randomly distributed 150 dots of 2° in a diameter were presented continuously on black background. The dots moved along 1 of 60 different directions and various speeds (7.94–403.2°/s with exponentially equal intervals) for 667 ms followed by a 833 ms interval during which dots were static. Color of each dot was set randomly in range of RGB (0,255,0) ∼ RGB (0,255,255) ∼ RGB (0,0,255). When dots overlapped, one was presented over the other in all experiments using random dots. When a dot went out of the screen, a new dot was created and appeared from the other edge of the screen at a random location with a random color. A different set of 100 random locations was used in each trial of the same visual area.

The order of all conditions of each stimulus type were shuffled and divided into 5 sessions. A session started with a gray screen (for gratings) or stationary dots for 5 sec to reduce onset transients. Each session was divided into 5 blocks interrupted by 5-s intervals of gray screen or stationary dots. As responses after a long interval tended to be larger, responses to the first stimuli after 5 s interval or the start were excluded for analysis. Sessions of different stimulus type were executed alternatively. Stimulus conditions were repeated twice.

### Quantification and statistical analysis

Data analysis was performed using custom-written script in LabVIEW (National Instruments) and MATLAB (Mathworks).

#### Image processing and data analysis

##### Wide-field autofluorescent imaging

ΔF/F value of each pixel was calculated as the mean pixel fluorescence value for 15 frames (starting 5 frames after stimulation onset) divided by the mean of 10 frames before the stimulation. The resulting ΔF/F values were averaged for all trials per stimulus condition and filtered with a square filter (5 × 5) to reduce shot noise (Figures 1B, 1C, S1B, and S1C).

##### 2-photon imaging

All collected frames of individual time-series were averaged (Figures 1D and S1D), and regions-of-interest (ROIs) were selected on the average image, where cell bodies were clearly identifiable. The ROIs were polygons drawn manually on the edge of the cell bodies to measure the intracellular calcium signal with minimal contamination by neuropil activity. The intensity values for all pixels in each ROI were averaged for each frame to obtain the raw temporal calcium signal of the respective cell. The distribution of calcium signal of each cell was Gaussian-fitted, and the center value was used as the baseline F. The normalized signal was differentiated and running averaged over 7 frames (3 frames before and 3 frames after each frame; Figure 1E). Positive values of the differentiated signal during the period in which a stimulus was presented were averaged, and the result was used as the response to the stimulus. The response values to the same stimulus were averaged and plotted in a response map for stimulus direction and speed/SF. All intensity values on the response map were plotted in a histogram to illustrate their distribution. An exponential Gaussian curve was then fitted to this distribution, and the response value corresponding to the maximum point of the fitted distribution curve was used as the background activity level for all analysis.

To determine how much neuropil contamination could affect our results, we performed the following analysis in a subset of cells following our previous procedure.^53^ For each ROI, we subtracted from its F the mean intensity of pixels in a 20 μm shell surrounding it^54^^,^^55^: F_Cell_ = F_ROI_ – r F_Neuropil_, where r, the neuropil contamination factor, is an estimate of the ratio of mean fluorescence present in small diameter blood vessels compared with the surrounding neuropil. We calculated r to be ∼0.3 in our study. Any cell whose neuropil shell encompassed a second cell, even partially, was excluded from this analysis. Our analysis indicated that neuropil subtraction did not cause any systematic or significant change in our measurements (data not shown). We thus did not subtract neuropil signal for the analysis in this study.

#### Selection criteria for analysis

For data analysis, we selected cells that showed certain level of activity, responsiveness, and selectivity using the following analyses.

##### Skewness

When a cell is active and the S/N is high enough, the distribution of calcium signal is skewed towards high value (Figures S1F–S1I). Then, cells whose skewness of signal distribution was larger than 0.5 were first selected for all analyses. Skewness was calculated as $skewness=\frac{\sqrt{n}\sum_{t=1}^{n}{\left(Ft-\bar{F}\right)}^{3}}{{\left(\sum_{t=1}^{n}{\left(Ft-\bar{F}\right)}^{2}\right)}^{3/2}}$, where Ft is fluorescent signal at each time point.

##### Responsiveness to stimulus

Stimulus conditions corresponding to the max response area (9 × 9 pixels) in the response map were selected, the center of which was the pixel with the max response (Figures S1J and S1K, orange square). Calcium signal transients between 0.2 sec before- and 1 sec after the start point of these selected stimuli were selected and averaged in each time bin (0.2 sec). Averaged signal intensities of the selected trials were then combined (Figure S1L). Differences in the signal intensities over the time bins were tested by a Friedman test. Neurons with p-values <0.05 were selected as responsive to visual stimulus for subsequent analyses.

##### Reliability and selectivity for specific stimulus conditions

We calculated the Pearson correlation coefficient of all pairs of adjacent pixels (4 sides) in the response map (Figures 2E–2G), and neurons with p < 0.05 were selected for 2D-fitting (Figure 1J) and motion-streak analysis (Figures 3E–3G and 4E–4H).

##### Statistically selective to certain directions

For comparison between moving dot axis and grating orientation (Figures 3E–3G and 4E–4H), we selected neurons statistically selective for specific motion directions to avoid noisy tuning curves. 5 rows of grating direction tuning curves (or 7 rows for moving dots) in the non-filter response map containing the peak point at the center row were selected (Figures S1K and S1M, red dashed boxes). The correlation of all pairs within 2 (or 3 for dots) columns (Figure S1M, white arrows) was calculated. Neurons with the p-value of correlation <0.05 were used for analysis. If the peak point fell at the edge of the map, we limited size of rows for this analysis.

##### Cell selection criteria

With these measures, we first used a minimal criteria (skewness >0.5, responsive p < 0.05) to select as many neurons as possible for the analysis of basic visual response properties (gOSI, gDSI, response reliability). The second criteria were used to select cells for tuning width analysis. We used 2D fitting for this purpose and only cells what were well fitted to the data (correlation coefficients >0.75) were included. The third criterion was used to select cells for motion streak analysis. We used preferred speeds and compared preferred motion axes for gratings and moving dots. To select cells with a clear preferred speed, cells with the correlation coefficient of a Gaussian fitting >0.75 were used. Criterion of gOSI >0.1 was then used to select cells with a clear orientation selectivity. The table below summarizes criteria used for individual analyses and corresponding figures. The proportions of neurons meeting these criteria were shown in Figures 1H–1J.

**Table:** 

Analysis	gOSI, gDSI, reliability	2D fitting	Motion Streak
Figure	Figures 2C, 2D, and 2G	Figure 2J	Figures 3E–3G and 4E–4H
Skewness	>0.5	>0.5	>0.5
Responsive to visual stimulus	p < 0.05	p < 0.05	p < 0.05
Correlation efficient between adjacent pixels		p < 0.05	p < 0.05
Correlation coefficient Data vs 2D model		r > 0.75	
Correlation coefficient Data vs Gaussian for speed			r > 0.75
gOSI for both dots and grating at peak points			>0.1
Statistically selective to certain direction at peak points			p < 0.05

##### gOSI, gDSI and preferred orientation/motion axis

The raw response map was smoothed with a square filter (5 × 5 for gratings, 7 × 7 for moving dots), and the pixel with the max value was determined. The row containing the max response point of a filtered response map was used for calculating gOSI, gDSI, and preferred motion-axis/orientation/direction and making the polar plot (Figure 2B). gOSI and gDSI of drifting grating or Global Motion-Axis Index (gASI) of moving dots was calculated by the vector sum method: gOSI (or gASI) = ((ΣR(*θi*)sin (2*θi*))^2^ + (ΣR(*θi*)cos(2θ*i*))^2^)^−2^/ΣR(*θi*), gDSI = ((ΣR(*θi*)sin (*θi*))^2^ + (ΣR(*θi*)cos(θ*i*))^2^)^−2^/ΣR(*θi*), where θ is the direction and R(*θ*) is the response to each stimulus direction. Values of R (*θ*) < 0 were replaced by R(*θ*) = 0. Preferred motion-axis (*θ*_prefAx_) and orientation (*θ*_prefOri_), were calculated from the vector sum: *θ*_prefAx_ = 1/2·arcsin(ΣR(*θi*)sin(2*θi*)/ΣR(*θi*)cos(2θ*i*)); *θ*_prefOri_ = *θ*_prefAx_ + 1/2·π.; Δaxis = |*θ*_prefAx.dot_ - *θ*_prefOri_|, where *θ*_prefAx.dot_ is preferred motion-axis of moving dots.*θi*

##### 2-D model fitting

A response map to drifting grating was fitted by a two-dimensional model composed of double von Mises curve (Swindale, 1998) for direction and Gaussian curve for SF. The 2-D model was: R(sf, *θ*) = (A_prf_·exp(k((cos *θ*- *θ*_prf_)-1)) + A_opp_·exp(k((cos *θ*- *θ*_prf_+π)-1)))· exp(-(*sf*- *sf*_prf_)^2^/c), where 1.3863<k<31.72 (for limits of half width of tuning curve at half-height *θ*_half_: 90°> *θ*_half_> 12°), *sf* is the log of SF, *sf*_prf_= preferred SF, *θ*_prf_= preferred axis, A_prf_ and A_opp_ are amplitude of two peaks of a double von Mises curve, A_prf_ > A_opp_> 0. Half width of tuning curve at half-height (Figures 2I and 2J) was calculated as: *θ*_half_ = arccos(1+ln0.5/k). The minimum limit of *θ*_half_ was set at 12° (the maximum limit of k = 31.72) to avoid that a small number of pixels determine a fitted 2D-model. When half-height width is 90°, values of responses at the direction perpendicular to the preferred axis will be a summation of half-height widths of two von Mises curves which can take a value close to the value at the preferred axis. Then, neurons well fitted with a 2D model with 60° >*θ*_half_>12° were selected for analysis. A 2D model was first fitted to the filtered response map to determine the initial values for the subsequent fitting to the non-filtered map. Neurons with a correlation-coefficient between the 2D model and the non-filtered map > 0.75 were selected for analysis in Figure 2J.

##### Preferred speed (or preferred SF)

7 columns of speed (or SF) tuning curves in the non-filtered response map to moving dots, the center of which contains the max response pixel, were selected (Figure S4B). The selected speed (or SF) tuning curves were averaged over directions, and fitted with a Gaussian (Figure 4D) as follows, R(v) = a·exp(-(v-v_max_)^2^/c^2^), where R(v) is the response, v is the log of speed (or SF), v_max_ is the log of the preferred speed (or SF) and its value was limited between 10-320°/s (0.02–0.32c/° for SF), a is the amplitude (a > 0), and c is the width of the tuning curve (2/3–3 octaves). Neurons with correlation coefficient between data and Gaussian >0.75 were selected for preferred speed or SF analysis. In the analysis for preferred speed at perpendicular- and parallel-axes in Figures 3E–3G, each cell was represented once as only one response peak was selected. However, some neurons exhibited both conventional motion-coding in slow-speed range and motion-streak coding in high-speed range (e.g., Figures 4B and S4A). To analyze both in the same cells, we selected a peak of the direction-tuning curve with Δaxis >60° (perpendicular axis) or <30° (parallel axis) separately. The largest peak point of direction tuning curves of moving dots at perpendicular- or parallel- axis was determined (Figures 4B and 4C). The 7 columns of speed tuning curves in the non-filtered map containing this peak point at the center column (Figure S4B) were analyzed using the method described above (Figure 4D). If there was no peak of direction tuning of moving dots at perpendicular- or parallel-axis, or the direction tuning curve at the peak did not show statistical selectivity for certain directions (detailed above), the neuron was excluded for analysis.

#### Statistics

Significance was calculated using one- or two-way ANOVA over all conditions in each visual area followed by Tukey HSD post-hoc test to determine differences between all combination of conditions in each visual area. For statistical analysis of speed and SF, the log values were used. Statistical significance was defined as a p-value <0.05. p-values and sample sizes are given in the results section or the figure.

## Data Availability

• All data reported in this paper will be shared by the lead contact upon request. • This paper does not report original code. • Any additional information required to reanalyze the data reported in this paper is available from the lead contact upon request.
