# Supplementary material for: Rapid development of motion-streak coding in the mouse visual cortex
Source: iScience. 2022 Dec 9;26(1):105778. doi: 10.1016/j.isci.2022.105778 (PMC9804142; doi:10.1016/j.isci.2022.105778)
Supplement: Document S1. Figures S1–S4 and Table S1 [file mmc1.pdf]

iScience, Volume *26*

Supplemental information

Rapid development of motion-streak coding in the mouse visual cortex

Manavu Tohmi and Jianhua Cang

Figure S1

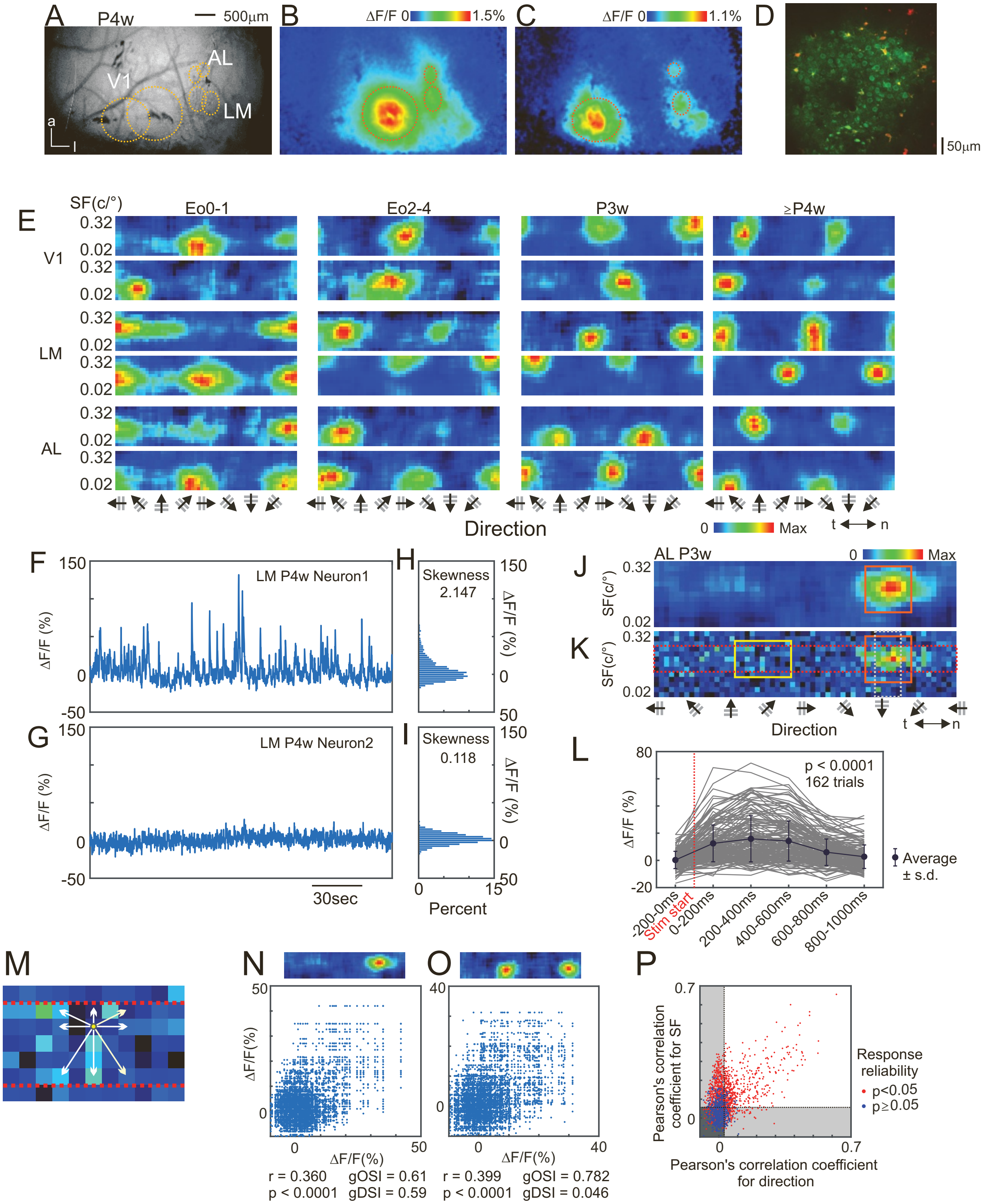

A
P4w
500μm
V1
AL
LM
a
l
B
ΔF/F 0
1.5%
C
ΔF/F 0
1.1%
D
50μm
E
SF(c/°)
Eo0-1
Eo2-4
P3w
≥P4w
V1
LM
AL
0.32
0.02
Direction
0
Max
t
n
F
LM P4w Neuron1
ΔF/F (%)
150
0
-50
G
LM P4w Neuron2
30sec
H
Skewness
2.147
I
Skewness
0.118
ΔF/F (%)
Percent
0
15
J
AL P3w
SF(c/°)
K
L
p < 0.0001
162 trials
ΔF/F (%)
80
-20
Average
± s.d.
-200-0ms
Stim start
0-200ms
200-400ms
400-600ms
600-800ms
800-1000ms
M
N
ΔF/F(%)
r = 0.360
p < 0.0001
gOSI = 0.61
gDSI = 0.59
O
r = 0.399
p < 0.0001
gOSI = 0.782
gDSI = 0.046
P
Pearson's correlation coefficient for SF
Pearson's correlation coefficient for direction
0.7
Response reliability
p<0.05
p≥0.05

Figure S1. Identification of mouse V1 and higher visual areas at P4w by wide-field imaging and examples of response tuning maps at different developmental stages, related to Figure 1 and 2

(A-C) Autofluorescence imaging of visual cortical areas at P4w. (A) Non-processed autofluorescence image of cortical surface. (B) and (C) Images of autofluorescence signal changes evoked by drifting gratings placed at different visual field positions, 45° azimuth in (B) and 90° azimuth in (C). Dashed ellipses represent corresponding locations in the 3 images.

(D) An image of area AL at P4w under 2-photon microscope. Green: cal-520 signal; red: SR-101 stained astrocytes.

(E) Examples of visual responses of V1, LM, and AL neurons at different developmental stages. See Figure 1G for other examples.

(F–I) Criteria to select active neurons. When a cell is active and the S/N is high, the distribution of calcium signal is skewed towards high value. (F) and (G): Examples of calcium transients of an active neuron (F) and a non-active neuron (G) in LP at P4w. (H) and (I): Distributions of signal intensities in F and G. The distribution in H is more skewed than in I. The skewness of each neuron was calculated (see Methods) and those with skewness > 0.5 were selected as active neurons for subsequent analyses.

(J-K) Examples of filtered (J) and non-filtered (K) response maps of an AL neuron for drifting grating at P3w. Orange square: the area the center of which is the max response pixel of the filtered map (9 x 9 pixels; panel E) is used to test the responsiveness to the visual stimuli (L). Dotted boxes: the areas are used to test selectivity for direction (red, 5 rows) or SF (or speed of moving dots; gray, 5 columns).

(L) Method to select neurons responsive to visual stimuli. Calcium transients of the max response area (orange boxes in J and K) between 0.2 s before and 1sec after stimulus start of these selected stimulus conditions were divided into 6 time-bins of 0.2 s each. Signal intensities in each time bin were averaged (gray lines). Friedman test was applied to test the difference in signal intensities over the time-bins. Neurons with $p<0.05$ were selected as responsive neurons for subsequent analyses. Blue line: Average of signal intensities. Error bar: s.d.

(M–O) Method to select neurons statistically selective to certain directions or SFs/speeds. (M) Magnification of the yellow box in K. To calculate Δaxis (Figures 3E-G) or perpendicular- or parallel-axes (Figures 4E-G), we selected neurons statistically selective for stimulus directions and speeds. 5 rows (or 5 columns for SF, gray dotted box in K) of grating direction tuning curves (or 7 rows/columns for moving dots), the center of which contains the max-response pixel, were selected (the red dashed boxes in K and M). Each pixel in the selected rows (for instance, the pixel with yellow dot in M) was compared with all pixels within 2 columns (or 2 rows for SF) in the selected rows (the range of white arrows in M; 3 columns/rows for dots). Correlation of signal intensities of all these pairs were plotted (N) and Pearson's correlation coefficient r and p value calculated. Note that “statistically selective to certain directions” is not the same as conventional “direction selectivity”. A classical “orientation selective” neuron (i.e., low gDSI and high gOSI; example in panel O) can show selectivity in this method. Neurons with $p < 0.05$ were used for analysis in Figures 3E-G and 4E-G.

(P) Correlation between selectivity for directions and SFs and response reliability of V1 neurons at Eo0-1. X- or Y-axis: correlation coefficient of direction (X) or SF (Y) selectivity test (M-O). Red or blue dot: cells which are reliably responsive (red) or non-responsive (blue) to certain stimulus conditions (Figure 2F) with $p<0.05$ ($p>=0.05$ for blue). Gray area: neurons are non-selective for either direction or SF (light gray) or both (dark gray) with $p>=0.05$. Many cells were only selective to either SF or direction.

Figure S2

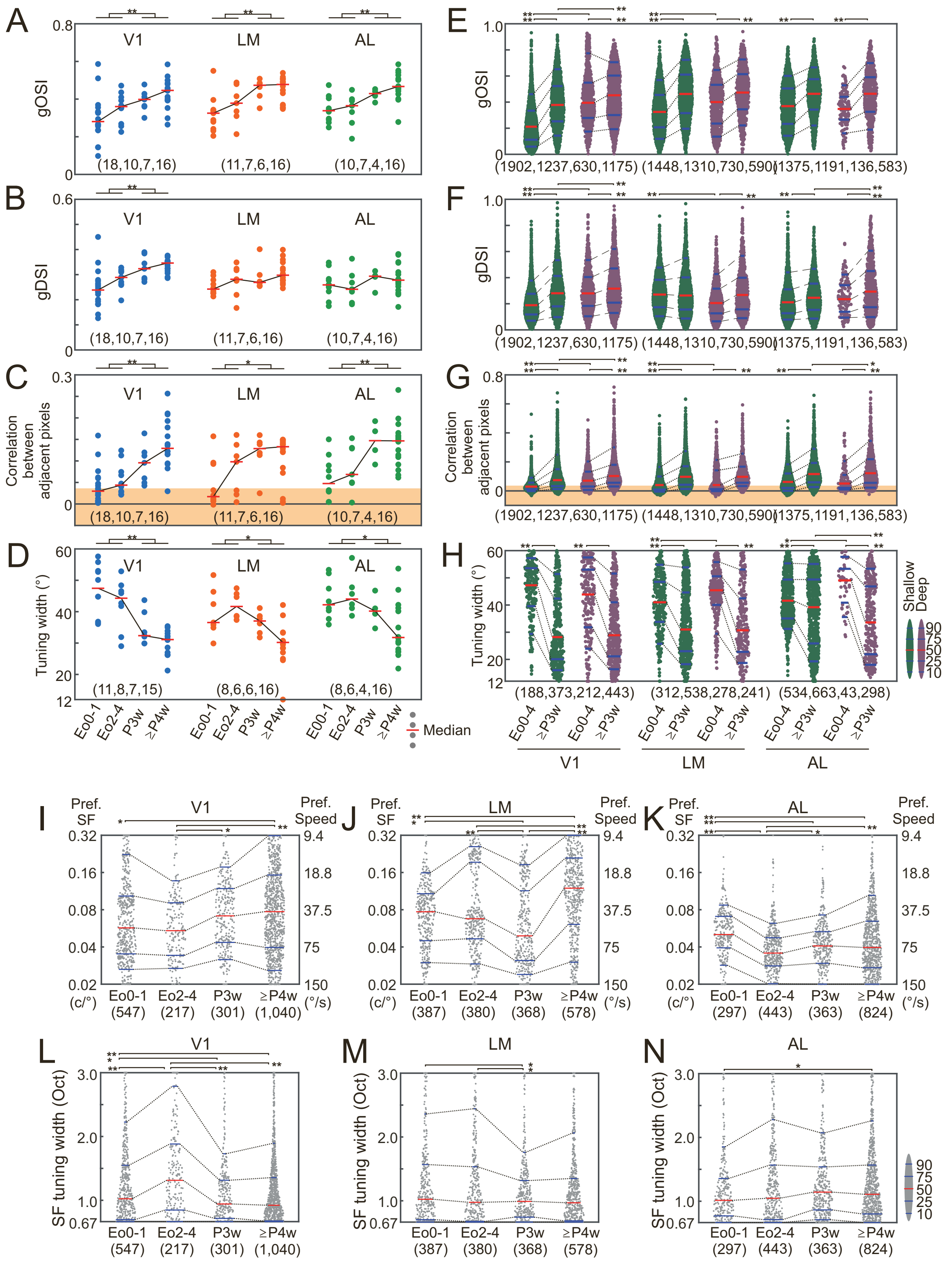

A
B
C
D
E
F
G
H
I
J
K
L
M
N
V1
LM
AL
gOSI
gDSI
Correlation between adjacent pixels
Tuning width (°)
(18,10,7,16)
(11,7,6,16)
(10,7,4,16)
(11,8,7,15)
(8,6,6,16)
(8,6,4,16)
(1902,1237,630,1175)
(1448,1310,730,590)
(1375,1191,136,583)
(188,373,212,443)
(312,538,278,241)
(534,663,43,298)
Eo0-1
Eo2-4
P3w
≥P4w
Eo0-4
≥P3w
Median
Shallow
Deep
Pref. SF
Pref. Speed
(c/°)
(°/s)
(547)
(217)
(301)
(1,040)
(387)
(380)
(368)
(578)
(297)
(443)
(363)
(824)
SF tuning width (Oct)

Figure S2. Criteria to select neurons for individual analyses, related to Figure 2

(A-D) Animal-to-animal variabilities of gOSI (A, data from Figure 2C), gDSI (B, Figure 2D), response reliabilities (C, Figure 2G), and tuning widths (D, Figure 2J). Each dot represents averaged data of each animal. Eo0-1 and Eo2-4, and P3w and >P4w were combined for statistical analysis to compensate for the small number of recordings in some conditions. There were significant differences between Eo0-4 and >=P3w in all areas for all analysis ($p < 0.05$ by Tukey HSD post-hoc test following two-way ANOVA, age x area, age: $p < 0.05$ for all areas) except for gDSIs in LM and AL ($p = 0.119$ and 0.659). *: $p<0.05$, **: $p<0.001$. See Table S1 for results of statistical analysis.

(E-H) Evaluation of the effects of recording depth on the results of Figure 2C, D, G, and J. We recorded neural activities from the depth of 150-350 μm from the brain surface which presumably corresponded to the layer 2/3 in adult mice (see Methods). However, since the facts that depth of -350 μm might be around the border between layers 3 and 4, and that the cortex around eye-opening is thinner than adults, we cannot rule out the possibility that some recordings were made at different layers of layer 2/3 where neurons possibly have different visual properties (de Vries et al., 2020). To address this point, we separated data into two groups, ones from recording sites above (shallow site, green dots) or below (deep site, purple) -280 μm from the brain surface and analyzed them separately. Eo0-1 and Eo2-4, and P3w and >P4w were combined to compensate for the small number of recordings in some conditions. The developmental changes that gOSIs, gDSIs, and response reliabilities increased (Figures 2C, D, and G) and that tuning width for directions sharpened (Figure 2J) were reproduced in all conditions (Eo0-4 vs >=P3w, $p<0.0001$, Tukey HSD post-hoc test following two-way ANOVA, depth x age, age: $p<0.0001$ for all area and depth) except for gDSI of shallow sites in LM ($p=0.408$). The light orange areas in C and G represent statistically non-significant correlation between adjacent pixels ($p>0.05$, $r < 0.036$, 3,000 pairs, Pearson's test).

(I–K) Distribution of preferred SFs of neurons in V1 (I), LM (J), and AL (K) over development. Since temporal frequency was fixed at 3Hz, the speed (right axis) is changed with the SF (left axis). There were no consistent tendencies in preferences for SFs over development in each area except for that preferred SFs of AL were lower than V1 and LM at all ages (AL vs V1 or LM at all ages, $p<0.0001$, Tukey HSD post-hoc test, following two-way ANOVA of age x area, age: $p<0.0001$, area: $p<0.0001$, interaction: $p<0.0001$).

(L–N) Distribution of SF tuning widths of neurons in V1 (L), LM (M), and AL (N), calculated by Gaussian fitting (see Figure 4D). σ of the Gaussian is used as tuning width. We analyzed active cells (see Figure S1Q-S) well fitted to the Gaussian with $r > 0.75$ of correlation between data and the gaussian in I-N. There were statistical differences between ages in all areas ($p<0.05$ for all areas, one way ANOVA). *: $p<0.05$, **: $p<0.001$, Tukey HSD post-hoc test. Blue bars in I-N: 10, 25, 75, and 90 percentiles of the entire populations. Red bars in I-N: median.

Figure S3

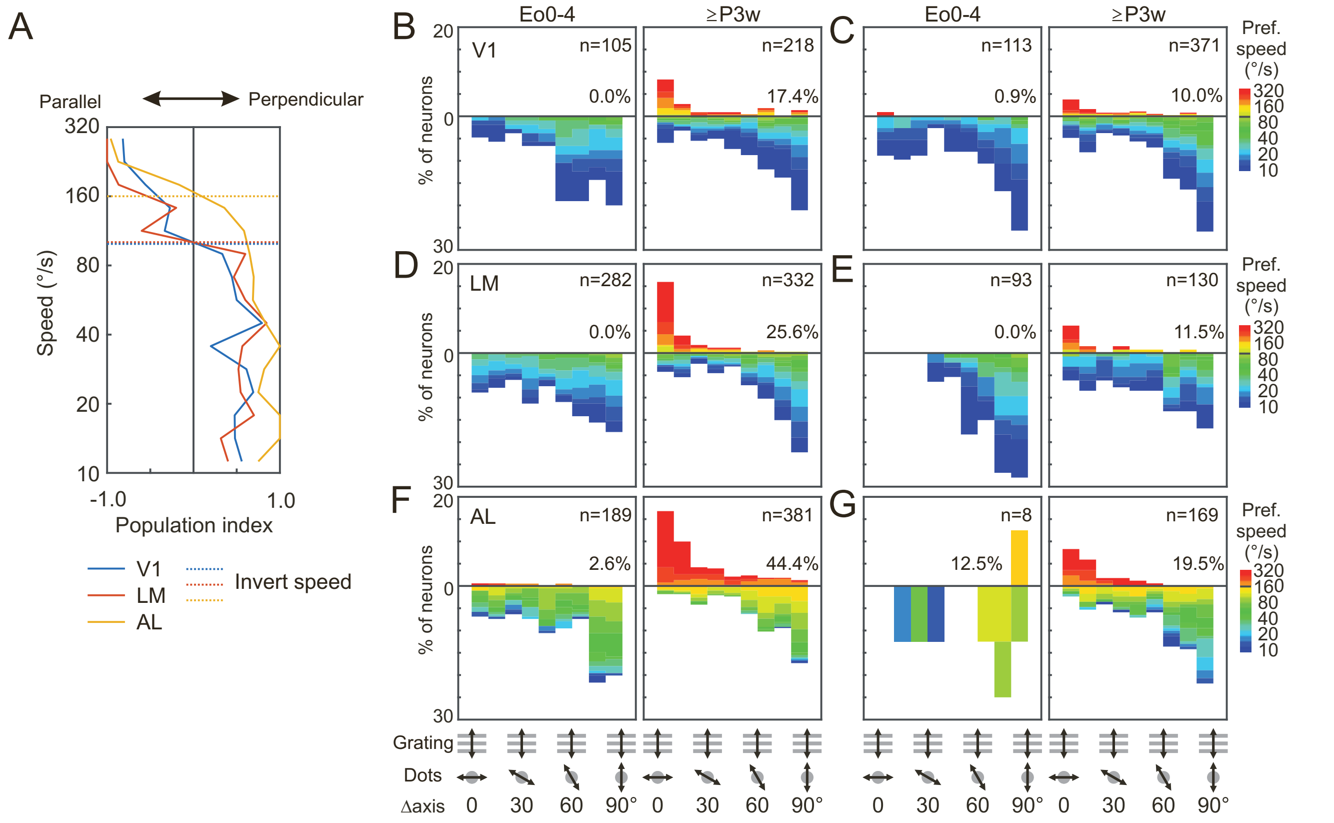

Figure S3. Determination of invert speed and analysis for depth, related to Figure 3
(A) We analyzed the data in the later development stages (P3w and >P4w were combined, the right two panels in Figures 3E-G) to determine the speed above which there are more cells encoding motion-streak than those encoding conventional motion. Populations of neurons with Δaxis > 60° ($N_{perpendicular}$) and < 30° ($N_{parallel}$) in each speed range were compared by calculating Proportion Index: ($N_{perpendicular} - N_{parallel}$)/ ($N_{perpendicular} + N_{parallel}$). The index is negative if the proportion preferring parallel axis (i.e, motion streak) is larger than that preferring perpendicular axis (i.e., conventional coding). Proportion indices were plotted as a function of speed in V1 (light-blue line), LM (orange), and AL (yellow). Dashed lines represent invert speeds (100.8°/s for V1 and LM, and 160°/s for AL) between upward and downward of bar graphs in Figures 3E-G.
(B-G) Evaluation of the effects of recording depth on the results of Figure 3E-G. Same convention as in Figure 3E-G, for V1 (B,C), LM (D,E), and AL (F, G) at different ages (left panels: Eo0-4, right panels: >=P3w) from different depth (B, D, F: shallow recording site, depth >-280μm; C, E, G: deep recording site, depth<=-280μm). Eo0-1 and Eo2-4, and P3w and >P4w are combined to compensate for the small number of recordings in some conditions. Proportions of neurons with preferred speeds higher than the invert speeds were different between ages ($p < 0.001$, chi-square test) at both depths in all areas except for ones at Eo0-4 in AL from deep sites (G) with the small number of cells ($n = 8$ at age of Eo0-4). Overall, the same developmental trend is seen for both shallow and deep recording.

Figure S4

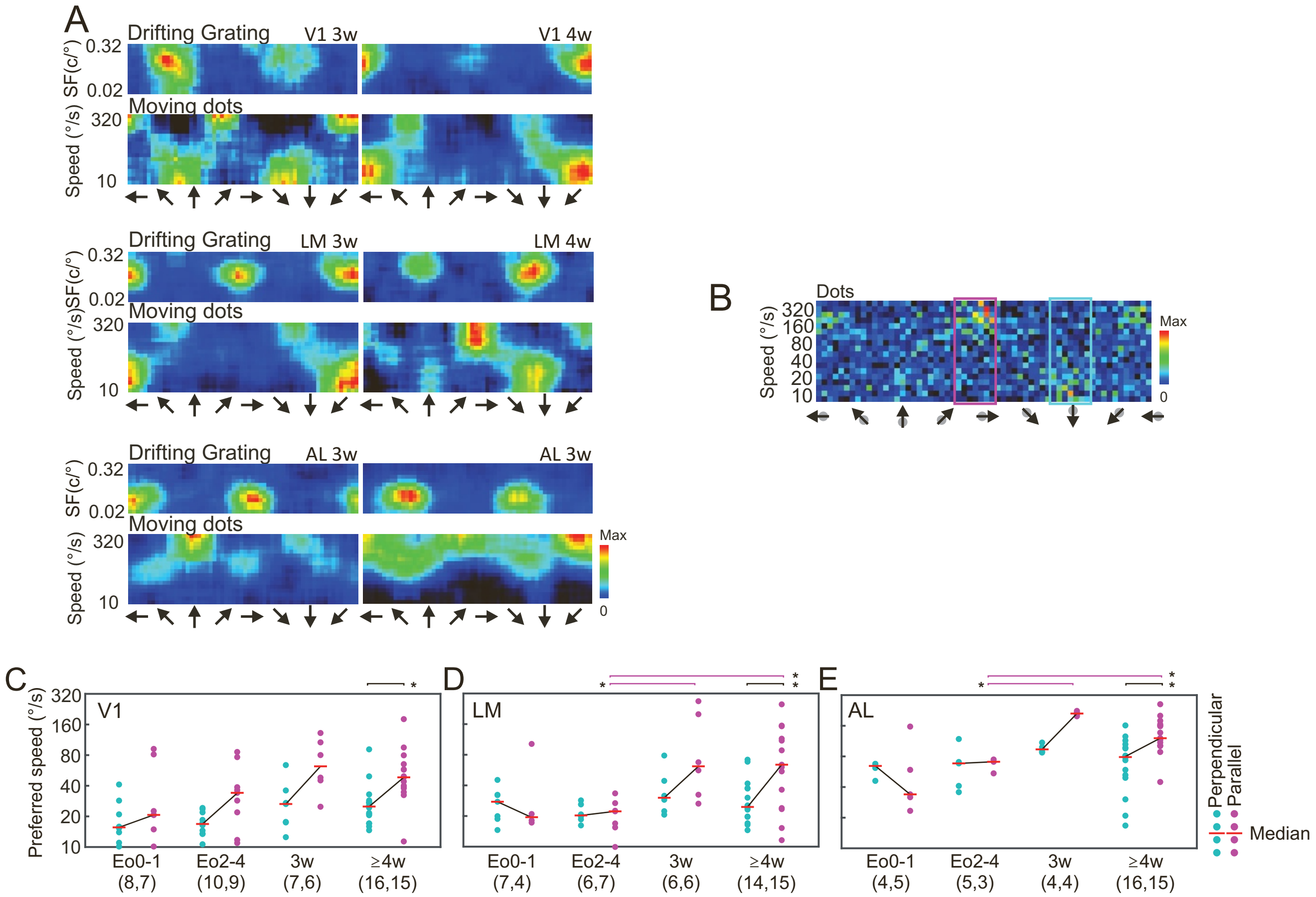

Figure S4. Analysis of transition cells, related to Figure 4

(A) Examples of transition cells, which showed conventional motion coding in slow speeds and motion streak coding in high speeds. See Figures 4A and B for another example.

(B) To calculate preferred speed at perpendicular- or parallel-axes (Figures 4E-G), a portion of the non-filtered response map was selected. 7 columns of speed tuning curves (light blue box for perpendicular axis; or pink box for parallel axis) in the non-filtered response map containing the selected peak (blue or red stars in B) in the center column were averaged over directions at each speed. The averaged responses (Figure 4D, blue and purple dots) were used for Gaussian fitting to determine the preferred speed.

(C–E) Animal-to-animal variabilities of preferred speeds of moving dots along perpendicular-(light blue) or parallel-(pink) axis of V1 (C), LM (D), and AL (E) neurons at different developmental stages. Each dot represents averaged data of each animal. *: $p < 0.05$ tested by Tukey HSD post-hoc test, following two-way ANOVA of axis x age, axis: $p<0.05$, age: $p<0.05$, interaction: $p>0.05$ for all areas. See Table S1 for resuts of statisticalanalysis.

Table S1. Statistical evaluation of Figure S2A-D and S4C-D, related to Figure 2 and 4

	Area	Age	Axis	Average	N	s.d	s.e	Comparison	Difference	F test (p-value)	T test (p-value)
gOSI (Fig.S2A)	V1	Eo0-4	-	0.317	28	0.109	0.021	ages	0.102	0.084	0.000
		>=P3w	-	0.419	23	0.076	0.016				
	LM	Eo0-4	-	0.342	18	0.097	0.023	ages	0.108	0.078	0.000
		>=P3w	-	0.450	22	0.064	0.014				
	AL	Eo0-4	-	0.343	17	0.075	0.018	ages	0.117	0.730	0.000
		>=P3w	-	0.460	20	0.069	0.016				
gDSI (Fig.S2B)	V1	Eo0-4	-	0.258	28	0.067	0.013	ages	0.075	0.002	0.000
		>=P3w	-	0.333	23	0.034	0.007				
	LM	Eo0-4	-	0.261	18	0.047	0.011	ages	0.042	0.993	0.007
		>=P3w	-	0.303	22	0.047	0.010				
	AL	Eo0-4	-	0.258	17	0.057	0.014	ages	0.026	0.828	0.162
		>=P3w	-	0.284	20	0.054	0.012				
Local correlation (Fig.S2C)	V1	Eo0-4	-	0.047	28	0.040	0.008	ages	0.079	0.081	0.000
		>=P3w	-	0.126	23	0.057	0.012				
	LM	Eo0-4	-	0.056	18	0.057	0.013	ages	0.053	0.649	0.004
		>=P3w	-	0.109	22	0.052	0.011				
	AL	Eo0-4	-	0.069	17	0.051	0.012	ages	0.071	0.956	0.000
		>=P3w	-	0.140	20	0.050	0.011				
Tuning width (Fig.S2D)	V1	Eo0-4	-	44.8	18	8.273	1.950	ages	13.413	0.019	0.000
		>=P3w	-	31.4	22	4.792	1.022				
	LM	Eo0-4	-	40.8	13	6.147	1.705	ages	9.240	0.841	0.000
		>=P3w	-	31.5	22	6.558	1.398				
	AL	Eo0-4	-	44.6	14	6.309	1.686	ages	8.789	0.320	0.002
		>=P3w	-	35.8	20	8.278	1.851				
Preferred speed (Fig.S4C-E)	V1	Eo0-4	PP	16.8	18	*0.517	*0.122	axes (Eo0-4)	*0.749	0.006	0.018
			PL	28.3	16	*1.06	*0.264	axes (>=P3w)	*1.030	0.257	0.000
		>=P3w	PP	24.8	23	*0.667	*0.139	ages (PP)	*0.557	0.287	0.005
			PL	50.6	21	*0.856	*0.187	ages (PL)	*0.838	0.372	0.015
	LM	Eo0-4	PP	23.5	13	*0.464	*0.129	axes (Eo0-4)	*-0.0538	0.051	0.846
			PL	22.7	11	*0.849	*0.256	axes (>=P3w)	*1.137	0.010	0.002
		>=P3w	PP	29.7	20	*0.712	*0.159	ages (PP)	*0.338	0.131	0.109
			PL	65.4	21	*1.318	*0.288	ages (PL)	*1.528	0.154	0.000
	AL	Eo0-4	PP	60.3	9	*0.504	*0.168	axes (Eo0-4)	*-0.172	0.169	0.613
			PL	53.6	8	*0.847	*0.299	axes (>=P3w)	*0.973	0.158	0.000
		>=P3w	PP	70.9	20	*0.829	*0.185	ages (PP)	*0.232	0.152	0.362
			PL	139.2	19	*0.591	*0.136	ages (PL)	*1.38	0.208	0.002

PP: Perpendicular axis, PL: Parallel axis, *: the unit is octave
